# Inhibition of peripheral macrophages by nicotinic acetylcholine receptor agonists suppresses spinal microglial activation and neuropathic pain in mice with peripheral nerve injury

**DOI:** 10.1186/s12974-018-1133-5

**Published:** 2018-03-27

**Authors:** Norikazu Kiguchi, Daichi Kobayashi, Fumihiro Saika, Shinsuke Matsuzaki, Shiroh Kishioka

**Affiliations:** Department of Pharmacology, Wakayama Medical University, 811-1 Kimiidera, Wakayama city, Wakayama 641-0012 Japan

**Keywords:** TC-2559, Sazetidine A, Neuroinflammation, Cytokine, Chemokine, Allodynia

## Abstract

**Background:**

Neuro–immune interaction underlies chronic neuroinflammation and aberrant sensory processing resulting in neuropathic pain. Despite the pathological significance of both neuroinflammation-driven peripheral sensitization and spinal sensitization, the functional relationship between these two distinct events has not been understood.

**Methods:**

In this study, we determined whether inhibition of inflammatory macrophages by administration of α4β2 nicotinic acetylcholine receptor (nAChR) agonists improves neuropathic pain and affects microglial activation in the spinal dorsal horn (SDH) in mice following partial sciatic nerve ligation (PSL). Expression levels of neuroinflammatory molecules were evaluated by RT-qPCR and immunohistochemistry, and PSL-induced mechanical allodynia was defined by the von Frey test.

**Results:**

Flow cytometry revealed that CD11b^+^ F4/80^+^ macrophages were accumulated in the injured sciatic nerve (SCN) after PSL. TC-2559, a full agonist for α4β2 nAChR, suppressed the upregulation of interleukin-1β (IL-1β) in the injured SCN after PSL and attenuated lipopolysaccharide-induced upregulation of IL-1β in cultured macrophages. Systemic (subcutaneous, s.c.) administration of TC-2559 during either the early (days 0–3) or middle/late (days 7–10) phase of PSL improved mechanical allodynia. Moreover, local (perineural, p.n.) administration of TC-2559 and sazetidine A, a partial agonist for α4β2 nAChR, during either the early or middle phase of PSL improved mechanical allodynia. However, p.n. administration of sazetidine A during the late (days 21–24) phase did not show the attenuating effect, whereas p.n. administration of TC-2559 during this phase relieved mechanical allodynia. Most importantly, p.n. administration of TC-2559 significantly suppressed morphological activation of Iba1^+^ microglia and decreased the upregulation of inflammatory microglia-dominant molecules, such as CD68, interferon regulatory factor 5, and IL-1β in the SDH after PSL.

**Conclusion:**

These findings support the notion that pharmacological inhibition of inflammatory macrophages using an α4β2 nAChR agonist exhibit a wide therapeutic window on neuropathic pain after nerve injury, and it could be nominated as a novel pharmacotherapy to relieve intractable pain.

**Electronic supplementary material:**

The online version of this article (10.1186/s12974-018-1133-5) contains supplementary material, which is available to authorized users.

## Background

Neuropathic pain evoked by damage to or dysfunction of the nervous system has a severe effect on quality of life because typical symptoms of neuropathic pain (i.e., spontaneous pain, hyperalgesia, and allodynia) are resistant to standard analgesics [1–3]. To clarify the pathophysiology of neuropathic pain, a number of experimental animal models have been developed, and several lines of evidence have identified key molecules involved in neuropathic pain [4, 5]. Importantly, neuro–immune interaction drives chronic neuroinflammation and aberrant sensory processing resulting in neuropathic pain. Upon nerve injury, damaged Schwann cells and tissue-resident macrophages produce soluble inflammatory cytokines, such as interleukin-1β (IL-1β) and tumor necrosis factor-α (TNFα), and chemokines, such as CC-chemokine ligand 2 (CCL2), CCL3, and CCL4, that recruit circulating leukocytes (i.e., macrophages, neutrophils, and lymphocytes) into the site of injury [6–10]. The crosstalk between neurons and immune cells through the cytokine–chemokine network is a fundamental component of neuropathic pain [11–13].

Among the major immune cells accumulated in the injured nerves [5, 14, 15], inflammatory-polarized macrophages play a pivotal role in the regulation of neuroinflammation [16–18] and function as a common peripheral regulator of neuropathic pain [8, 19, 20]. Indeed, depletion of macrophages by a macrophage-targeting toxin or blockade of macrophage-derived inflammatory cytokines and chemokines by selective inhibitors prevents diverse experimental neuropathic pain in rodents [6, 11, 13, 21]. Given that key molecules for neuropathic pain are derived from inflammatory macrophages, comprehensive regulation of macrophage polarization may largely affect the pathogenesis of neuropathic pain [22].

Nicotinic acetylcholine receptors (nAChRs), which are ligand-gated cation channels consisting of homo- or hetero-pentameric complex from distinct subunits [23, 24], are expressed on peripheral macrophages, and their ligands, including nicotine, improve a variety of intractable inflammatory diseases in rodents [25–27]. We have previously demonstrated that the α4β2 subtype of nAChR plays an important role in the suppression of inflammatory macrophages in injured nerves, and local administration of α4β2-selective agonists relieves neuropathic pain in rodents [28, 29]. These findings suggest that pharmacological approaches targeting macrophages may be beneficial for treating neuropathic pain.

Prolonged abnormal input from primary sensory neurons into the spinal dorsal horn (SDH) triggers central sensitization [30–32], defined by increased excitability of pain-processing neurons and activation of glial cells (i.e., microglia and astrocytes) [33, 34]. Notably, microglia are often activated by several neurotransmitters, including cytokines, chemokines, and nucleotides, released from primary afferents and spinal cells, and microglia have been the focus of research during past decades as critical regulators of spinal neuroinflammation in neuropathic pain [33–35]. Activated microglia produce a variety of proinflammatory factors, which directly or indirectly sensitize pain-processing neurons in the SDH. Like peripheral events, typical inflammatory cytokines, chemokines, and growth factors are upregulated in the SDH after peripheral nerve injury, and several reports have demonstrated that inhibition of these molecules reverses neuropathic pain [33–35].

Despite the pathological significance of both neuroinflammation-driven peripheral sensitization and spinal sensitization mediated by glial cells, the functional relationship between these two distinct events has not been clarified. In this study, we determined whether inhibition of inflammatory macrophages by peripheral administration of α4β2 nAChR agonists affects microglial activation and upregulation of microglial factors in the SDH, which underlies spinal sensitization and neuropathic pain in mice following partial sciatic nerve ligation (PSL).

## Methods

### Animals and surgery

All animal experiments were approved by the Animal Research Committees of Wakayama Medical University and were carried out in accordance with the in-house guidelines for the care and use of laboratory animals of Wakayama Medical University. Male ICR mice aged 4 to 5 weeks (SLC, Hamamatsu, Japan) were used in all experiments, which complied with the Ethical Guidelines of the International Association for the Study of Pain. Mice were housed in plastic cages in a temperature controlled room (23–24 °C, 60–70% humidity) with a 12-h dark/light cycle and provided with water and food ad libitum. To induce neuropathic pain, mice were subjected to PSL according to a well-characterized procedure [36]. Under isoflurane anesthesia, the common sciatic nerve (SCN) was exposed through a small skin incision on one side (ipsilateral). Approximately one third of the SCN thickness was tightly ligated with a silk suture, and then the incision was closed by suturing and sterilized with povidone–iodine. For the sham controls, the SCN was exposed but not ligated before the incision was closed.

### Flow cytometry

Mice were euthanized by decapitation, and the fresh SCN was collected. A single-cell suspension was prepared by digestion with 2 mg/ml of collagenase D (Roche, Basel, Switzerland) and 40 μg/ml of DNase I (Takara Bio Inc., Kusatsu, Japan) in Dulbecco’s modified Eagle’s medium (DMEM; Sigma-Aldrich, Tokyo, Japan) and incubated at 37 °C for 30 min followed by gentle dissociation through 35-μm cell strainers (BD Biosciences, San Jose, CA). Red blood cells were lysed by incubating the cells for 1 min with 150 mM NH_4_Cl, 10 mM KHCO_3_, and 1 mM EDTA containing buffer at room temperature. The cells were collected by centrifugation at 400×*g* for 5 min, resuspended in FACS buffer (phosphate-buffered saline (PBS) containing 0.1% bovine serum albumin (Wako, Osaka, Japan) and penicillin–streptomycin (P/S)). Subsequently, cells were blocked with 10 μg/ml normal mouse IgG (EMD Millipore, Burlington, MA) in FACS buffer for 20 min at room temperature and incubated with Brilliant Violet 421™-conjugated anti-F4/80 antibody (mouse monoclonal, 2.5 μg/ml, BioLegend, San Diego, CA) and Phycoerythrin-conjugated anti-CD11b antibody (mouse monoclonal, 2.5 μg/ml, BioLegend) in FACS buffer on ice for 20 min, followed by a rinse with FACS buffer. Samples were read using FACSVerse™ flow cytometer (BD Bioscience) for 2.5 min at high flow speed, and the data were analyzed using FlowJo v10 software (Tree Star Inc, Ashland, OR).

### Drug administration

TC-2559 difumarate (4-(5-ethoxy-3-pyridinyl)-*N*-methyl-(*3E*)-3-buten-1-amine difumarate), sazetidine A dihydrochloride (6-[5-[(*2S*)-2-azetidinylmethoxy]-3-pyridinyl]-5-hexyn-1-ol dihydrochloride), and dihydro-β-erythroidine hydrobromide ((2*S*,13b*S*)-2-methoxy-2,3,5,6,8,9,10,13-octahydro-1*H*,12*H*-benzo[*i*]pyrano[3,4-*g*]indolizin-12-one hydrobromide; DHβE) were purchased from Tocris Biosciences (Bristol, UK). These agents were dissolved in sterile water at a higher concentration and diluted with sterile PBS for use. Perineural injection was performed according to a method described previously [9, 37]. In brief, under isoflurane anesthesia, the agents (10 μl) were injected without a skin incision into the region surrounding the SCN, using a microsyringe fitted with a 30-gauge needle connected to a cannula. Injections of TC-2559, sazatidine A, or DHβE were administered consecutively for 4 days during the first week (days 0, 1, 2, and 3; early phase), second week (days 7, 8, 9, and 10; middle/late phase), or fourth week (days 21, 22, 23, and 24; much later phase) after PSL. Bupivacaine hydrochloride (Sigma-Aldrich) was dissolved in sterile saline at 0.5% concentration, which were administered for consecutive 6 days after PSL (days 0–5).

### Macrophage culture

To collect peritoneal macrophages from naïve mice, an incision was made in the peritoneal membrane and 3 ml of chilled sterile PBS containing 1% P/S was slowly injected into the peritoneal cavity. Collected macrophages after flushing were washed with PBS and then cultured in DMEM containing 10% fetal bovine serum (FBS) and 1% P/S at 37 °C in an atmosphere of 5% CO_2_. TC-2559 (Tocris Biosciences) and lipopolysaccharide (LPS; Sigma Aldrich) were dissolved in sterile PBS and diluted with DMEM for use. For the experiments, macrophages were seeded in a poly-l-lysine-coated 24-well culture dish. At least 3 h before experiments, the culture medium was changed to DMEM without FBS, and cells were incubated with LPS and TC-2559 for 24 h.

### Behavioral testing

For evaluating mechanical allodynia, the 50% withdrawal threshold was determined by the von Frey test in accordance with a previously established method [38]. Briefly, mice were individually placed on a 5 × 5-mm wire mesh grid floor and covered with an opaque acrylic box. After adaptation for 2 to 3 h, calibrated von Frey filaments (Neuroscience, Tokyo, Japan) were applied to the middle of the plantar surface of the hind paw through the bottom of the mesh floor. In the paradigm of the up–down method, testing was initiated with a 0.4 g force in the middle of the series (0.02, 0.04, 0.07, 0.16, 0.4, 0.6, 1.0, 1.4, and 2.0 g). Stimuli were always presented in a consecutive fashion, either ascending or descending. In the absence of a paw withdrawal response to the selected force, a stronger stimulus was applied. In the presence of paw withdrawal, the next weaker stimulus was chosen. According to Chaplan et al. [38], after the response threshold was first crossed (the two responses straddling the threshold), four additional stimuli were applied. Based on the responses to the series of the von Frey filament, the 50% paw withdrawal threshold was calculated.

### Immunohistochemistry

The SCN and lumbar spinal cord (L4–5) were collected from mice after transcardiac perfusion with PBS followed by 4% paraformaldehyde and was post-fixed in 4% paraformaldehyde and dehydrated in 30% sucrose at 4 °C overnight. Frozen tissues embedded in freezing compound (Sakura, Tokyo, Japan) were cut longitudinally into 10-μm-thick (SCN) or 30-μm-thick (spinal cord) sections with a cryostat and mounted on glass slides (SCN) or floated in PBS (spinal cord). The sections were treated with PBS containing 0.3% Triton X-100 (PBST) for 1 h and then blocked with 5% normal donkey serum in 0.3% PBST at room temperature for 2 h. The sections were incubated with primary antibodies against F4/80 (rat monoclonal, 1:200; Cederlane, Burlington, Canada), IRF5 (rabbit monoclonal, 1:250; Abcam, Cambridge, MA), Iba1 (rabbit polyclonal, 1:500; Wako), or CD68 (rat monoclonal, 1:100; Bio-Rad Laboratories, Hercules, CA) at 4 °C overnight. All antibodies were diluted in 1% normal donkey serum in 0.1% PBST. The following day, sections were rinsed in PBST and incubated with fluorescence-conjugated secondary antibodies (1:300; Abcam) at room temperature for 2 h. Sections were rinsed in PBST and then incubated with Hoechst 33342 (Thermo Fisher Scientific, Waltham, MA) at room temperature for 10 min. Finally, sections were air-dried on glass slides for 30 min and coverslipped with mounting medium. Fluorescence images of SDH were detected using a confocal laser scanning microscope (Carl Zeiss, Oberkochen, Germany). Fluorescent intensities of Iba1 and the number of Iba1-positive cells (microglia) were measured in an indicated square of 100 × 100 μm^2^ area using ImageJ software.

### RT-qPCR

Mice were euthanized by decapitation, and the fresh SCN and lumbar SDH (L3–4 or L4–5) were collected in RNAlater solution (Thermo Fisher Scientific). The TRIzol® Plus RNA Purification Kit (Thermo Fisher Scientific) was used for the isolation of total RNA from the tissues following the manufacturer’s instructions. Briefly, tissues were placed in a 1.5-ml RNase-free tube and homogenized with TRIzol reagent. Chloroform was added to each sample, which were then centrifuged at 4 °C for 15 min. The aqueous phase containing RNA was transferred to a fresh tube, and RNA was isolated by purification column. Total RNA extract was used for the synthesis of cDNA by reverse transcription as follows. Total RNA was incubated with Random Primers (Promega, Madison, WI) at 70 °C for 5 min and then was cooled on ice. Samples were converted to cDNA by incubation with M-MLV Reverse Transcriptase and dNTPMix (Promega) at 37 °C for 50 min. qPCR was performed using AriaMx Real-Time PCR System (Agilent Technologies, Santa Clara, CA) by using the cDNA as the template, primers for each gene (Thermo Fisher Scientific) and SYBR® Premix Ex Taq™ II (Takara Bio Inc.). The primer sequences are listed in Table 1. Reactions were performed under the following conditions: 3 min at 95 °C, followed by 45 cycles of two steps, 10 s at 95 °C, and 30 s at 60 °C. The fluorescence intensities were recorded, and data were normalized to glyceraldehyde-3-phosphate dehydrogenase (GAPDH) or β-actin (ACTB).

**Table 1:** Primer sequences for RT-qPCR

Gene	Forward (5′ to 3′)	Reverse (5′ to 3′)
GAPDH	GGGTGTGAACCACGAGAAAT	ACTGTGGTCATGAGCCCTTC
ACTB	CAGCTGAGAGGGAAATCGTG	TCTCCAGGGAGGAAGAGGAT
IL-1β	AAAGCTCTCCACCTCAATGG	AGGCCACAGGTATTTTGTCG
Iba1	ATGAGCCAAAGCAGGGATTT	TTGGGATCATCGAGGAATTG
CD11b	GTTTCTACTGTCCCCCAGCA	GTTGGAGCCGAACAAATAGC
CD68	ACTCATAACCCTGCCACCAC	CCAACAGTGGAGGATCTTGG
IRF5	ACACTGAAGGGGTGGATGAG	CGAGGGCCATCATAGAACAG
IRF7	GTGTGTCCCCAGGATCATTT	CTGCAGAACCTGAAGCAAGA
CCL3	CTGCCCTTGCTGTTCTTCTC	GTGGAATCTTCCGGCTGTAG

### Western blotting

Mice were euthanized by decapitation, and the fresh lumbar SDH (L4–5) was collected. The tissues were sonicated in SDS sample buffer (50 mM Tris, 10% glycerol, 2% SDS, pH 7.4), which were then centrifuged at 10 °C for 5 min. The supernatant was transferred to a fresh tube, and total protein concentration of the prepared extracts was measured using the Dc protein assay (Bio-Rad Laboratories). Thirty micrograms of protein extracts were electrophoresed in 10% Mini-PROTEAN® TGX™ Precast Gel (Bio-Rad Laboratories) and transferred to a polyvinylidene difluoride membrane. The membrane was blocked with 5% nonfat dried milk in TBS containing 0.1% Tween 20 (TBST) at room temperature for 2 h and incubated with primary antibodies against IRF5 (rabbit monoclonal, 1:500; Abcam) or ACTB (HRP-conjugated, 1:2000; MBL, Nagoya, Japan) at 4 °C overnight. The following day, membrane was rinsed in TBST and incubated with HRP-conjugated secondary antibody (1:2000; Thermo Fisher Scientific). Immunoreactive bands were detected using a chemiluminescence reagent (Wako) for the detection of HRP, and band intensities were analyzed using ImageJ software.

### Statistical analysis

Data are presented as mean ± SEM. Statistical analyses were performed using Student’s *t* test, one-way analysis of variance followed by Tukey’s multiple comparison test, or two-way analysis of variance followed by Bonferroni’s multiple comparison test as appropriate. Statistical significance was established at *P* < 0.05.

## Results

### Accumulation of macrophages in the injured SCN after PSL

**Fig. 1:**
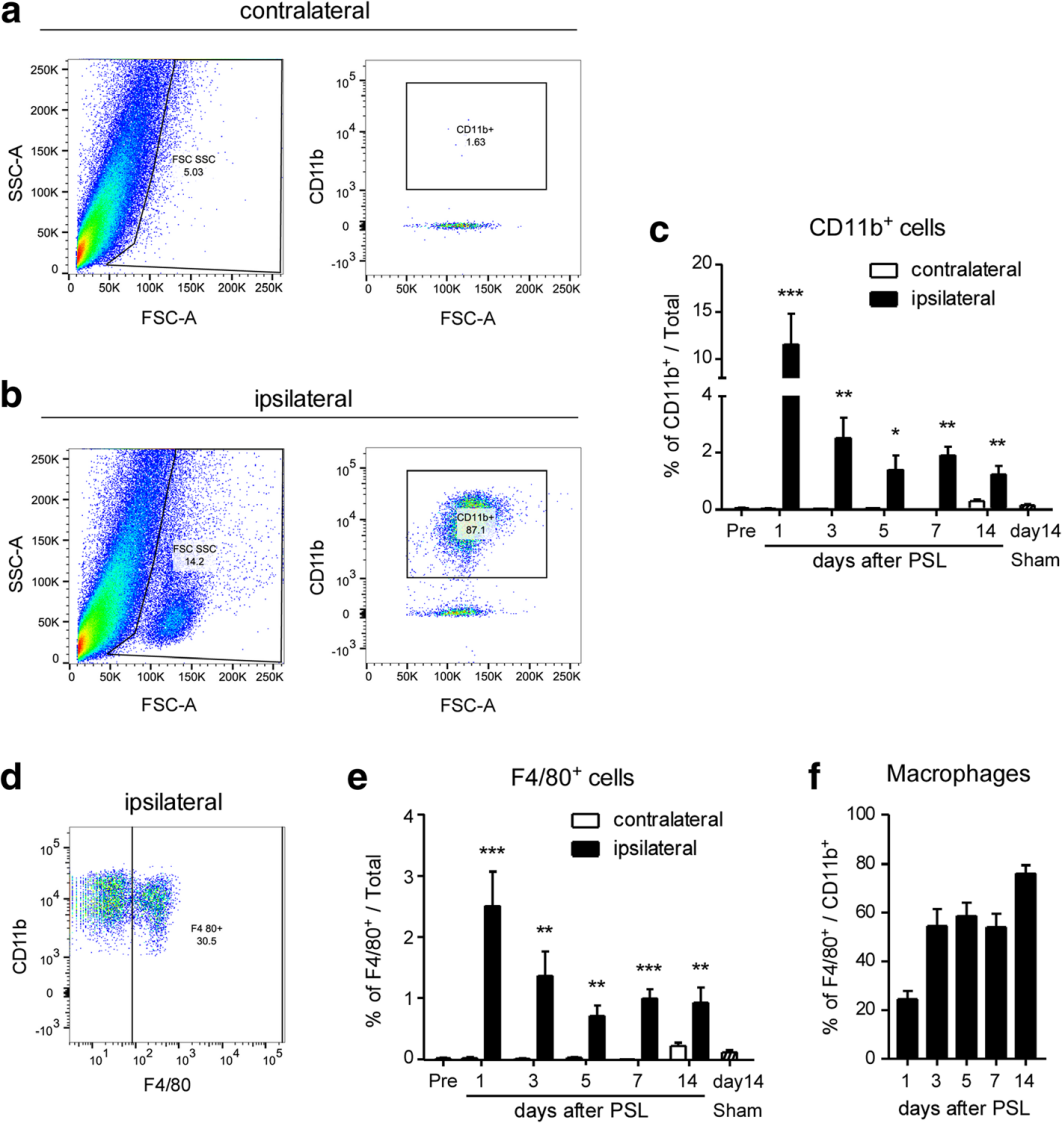
Accumulation of macrophages in the injured SCN after PSL. Mice were subject to partial sciatic nerve ligation (PSL) or sham surgery, and the sciatic nerve (SCN) was collected. Isolated cells from the SCN were analyzed by flow cytometry. Representative forward scatter (FSC-A; cell-surface area or size) versus side scatter (SSC-A; cell granularity or complexity) plots presenting total events collected from contralateral (**a**, undamaged) and ipsilateral (**b**, injured) SCN on day 1 after PSL are presented. Leukocyte gates used for subsequent analysis are shown (**a**, **b**). Representative FSC-A versus CD11b expression plot from leukocyte gate indicate the population of CD11b^+^ cells from total leukocytes in contralateral (**a**) and ipsilateral (**b**) SCN. **c**, **e** Quantitative analysis for the percentage of CD11b^+^ cells (**c**) and F4/80^+^ cells (**e**) in total events from intact (pre-injury), contralateral and ipsilateral SCN (PSL and sham) indicates the accumulation of CD11b^+^ leukocytes in the injured SCN on days 1 to 14. **d** Representative plot of F4/80 versus CD11b expression from leukocyte gate (**b**, right panels) in the ipsilateral SCN presents F4/80^+^ CD11b^+^ macrophages in the injured SCN on day 1 after PSL. **f** The percentage of F4/80^+^ macrophages in CD11b^+^ leukocytes are shown on days 1 to 14 after PSL. Data are presented as the mean ± SEM; *n* = 5–6. ***P < 0.001, **P < 0.01, *P < 0.05 versus pre-injury

To demonstrate that PSL recruits circulating monocytes/macrophages in the injured nerves, we performed flow cytometric analysis of CD11b (a broad marker for peripheral leukocytes and spinal microglia) and F4/80 (a specific marker for macrophages). Compared with the contralateral (undamaged) SCN, a distinct cell population was observed in the ipsilateral (injured) SCN on day 1 after PSL (Fig. 1a, b, left panels). The majority (87.1%) of this population in the ipsilateral SCN consisted of CD11b^+^ leukocytes, and the CD11b expression was markedly higher than that in contralateral SCN (Fig. 1a, b, right panels). The proportion of CD11b^+^ cells in the ipsilateral SCN was highest on day 1, and significant increases persisted for more than 2 weeks in comparison with the intact (pre-injury) SCN (Fig. 1c). Based on F4/80 expression, approximately 30% of CD11b^+^ cells were macrophages (CD11b^+^ and F4/80^+^) on day 1 after PSL, and all F4/80^+^ cells expressed CD11b (Fig. 1d). Similar to CD11b^+^ cells, the proportion of F4/80^+^ cells in the ipsilateral SCN was significantly higher than that in the intact SCN for more than 2 weeks (Fig. 1e). In contrast, neither CD11b^+^ nor F4/80^+^ cells were increased in the contralateral SCN during 2 weeks after PSL (Fig. 1c, e). The percentage of macrophages (CD11b^+^ and F4/80^+^) in total CD11b^+^ cells in the ipsilateral SCN increased to more than 50% from days 3 to 7, and reached 76.1% on day 14 after PSL (Fig. 1f).

### Inhibition of IL-1β overexpression in macrophages by TC-2559

**Fig. 2:**
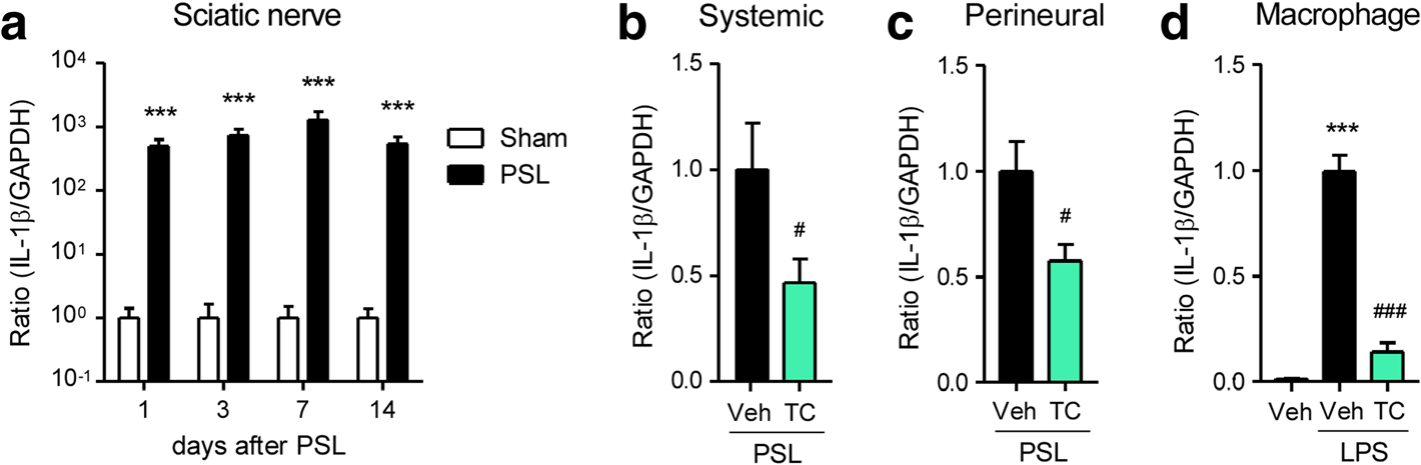
Inhibition of interleukin-1β (IL-1β) overexpression in macrophages by TC-2559. Mice were subject to PSL or sham surgery. TC-2559 was administered subcutaneously (s.c., 22.8 μmol/kg) or perineurally (p.n., 20 nmol) on days 0, 1, 2, and 3, and the SCN was collected. Expression levels of IL-1β mRNA were analyzed by RT-qPCR. **a** Time course of IL-1β mRNA expression in the SCN on days 1 to 14 after sham or PSL. Inhibition of PSL-induced upregulation of IL-1β mRNA in the SCN on day 7 after PSL by s.c. (**b**) or p.n. (**c**) administration of TC-2559. **d** Peritoneal macrophages collected from naïve mice were treated with lipopolysaccharide (LPS, 50 ng/ml) and TC-2559 (0.5 mM), and cells were incubated for 24 h. Inhibition of LPS-induced upregulation of IL-1β mRNA by TC-2559 is shown. Data are presented as the mean ± SEM; *n* = 5–12. ***P < 0.001 versus sham. ^###^*P* < 0.001, ^#^*P* < 0.05 versus PSL/Veh or LPS/Veh

To determine if the α4β2 nAChR agonist inhibits inflammatory macrophages, we evaluated the mRNA expression levels of IL-1β by RT-qPCR. The mRNA levels of IL-1β were upregulated several hundred folds in the injured SCN after PSL, and the upregulation persisted for more than 2 weeks (Fig. 2a). Upregulation of IL-1β mRNA in the injured SCN on day 7 after PSL was suppressed by either systemic (subcutaneous, s.c.) or local (perineural, p.n.) administration of TC-2559 once a day for 4 days (days 0–3; Fig. 2b, c). In cultured peritoneal macrophages, mRNA expression level of IL-1β was markedly upregulated by LPS (50 ng/ml) treatment for 24 h and was attenuated by co-treatment of TC-2559 (0.5 mM; Fig. 2d).

### Improvement of mechanical allodynia after PSL by systemic TC-2559

**Fig. 3:**
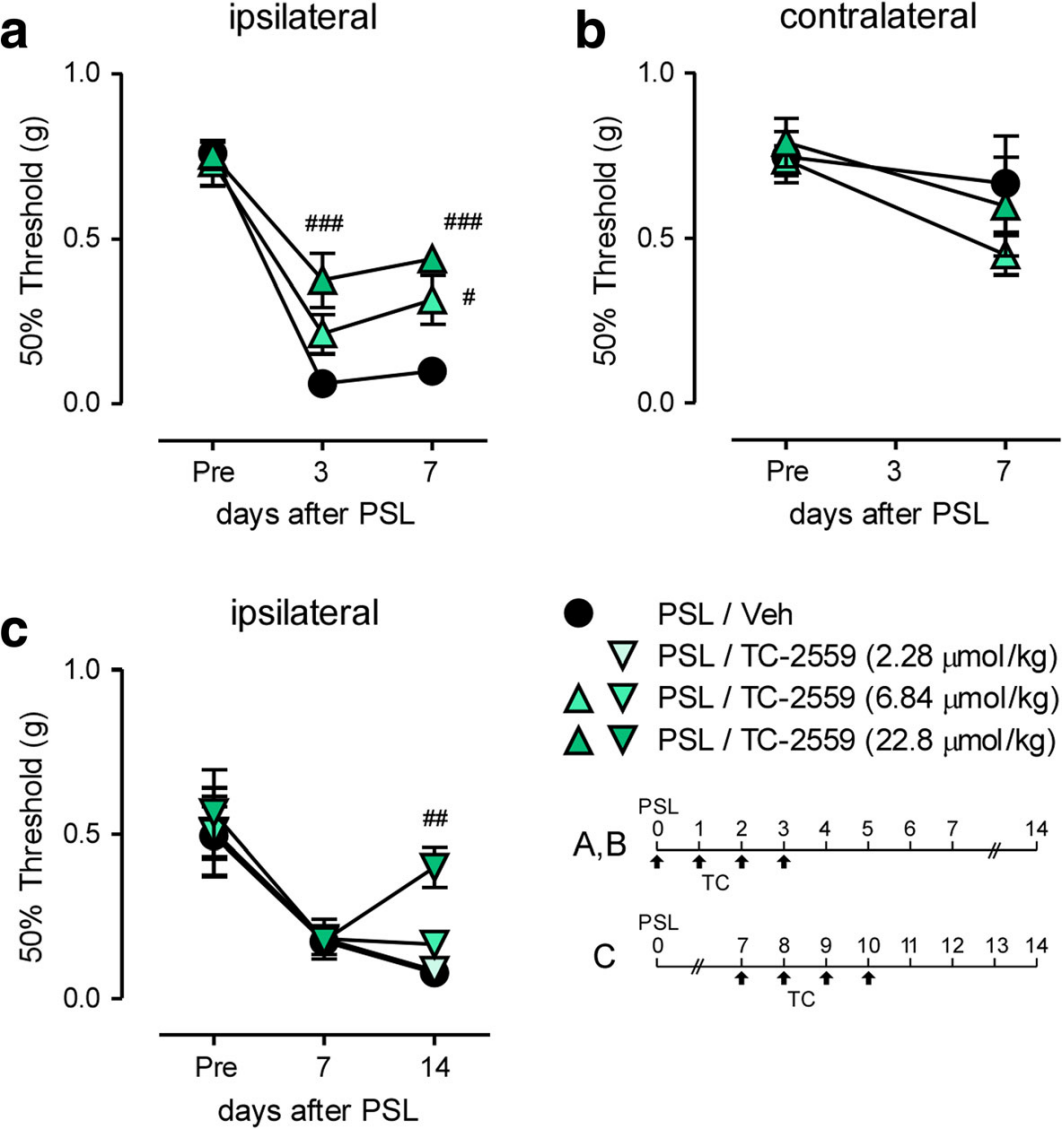
Improvement of mechanical allodynia after PSL by systemic TC-2559. Mice were subject to PSL, and TC-2559 was administered (s.c., 2.28–22.8 μmol/kg) four times according to the indicated schedules. The 50% paw withdrawal mechanical threshold was assessed by the up–down method using the von Frey test. **a** Attenuation of PSL-induced mechanical allodynia in the ipsilateral side by TC-2559 (days 0, 1, 2, and 3) are shown. **b** There was no significant difference in the contralateral side in all groups. **c** Improvement of PSL-induced mechanical allodynia in the ipsilateral side by TC-2559 (days 7, 8, 9, and 10) are shown. Data are presented as the mean ± SEM; *n* = 5–9. ^###^*P* < 0.001, ^##^*P* < 0.01, ^#^*P* < 0.05 versus PSL/Veh

We assessed whether systemic administration of TC-2559 affects neuropathic pain following PSL. On the ipsilateral side, the 50% withdrawal threshold evaluated by the von Frey test was markedly decreased on days 3 and 7, indicating mechanical allodynia. PSL-induced mechanical allodynia was significantly attenuated by s.c. administration of TC-2559 (6.84, 22.8 μmol/kg, days 0–3) (Fig. 3a). In contrast, no significant difference of the 50% withdrawal threshold was observed on the contralateral side (Fig. 3b). Moreover, PSL-induced mechanical allodynia was relieved by TC-2559 (22.8 μmol/kg, s.c.) even if it was administered four times on days 7–10 after PSL (Fig. 3c).

### Improvement of mechanical allodynia after PSL by peripheral nAChR agonists

**Fig. 4:**
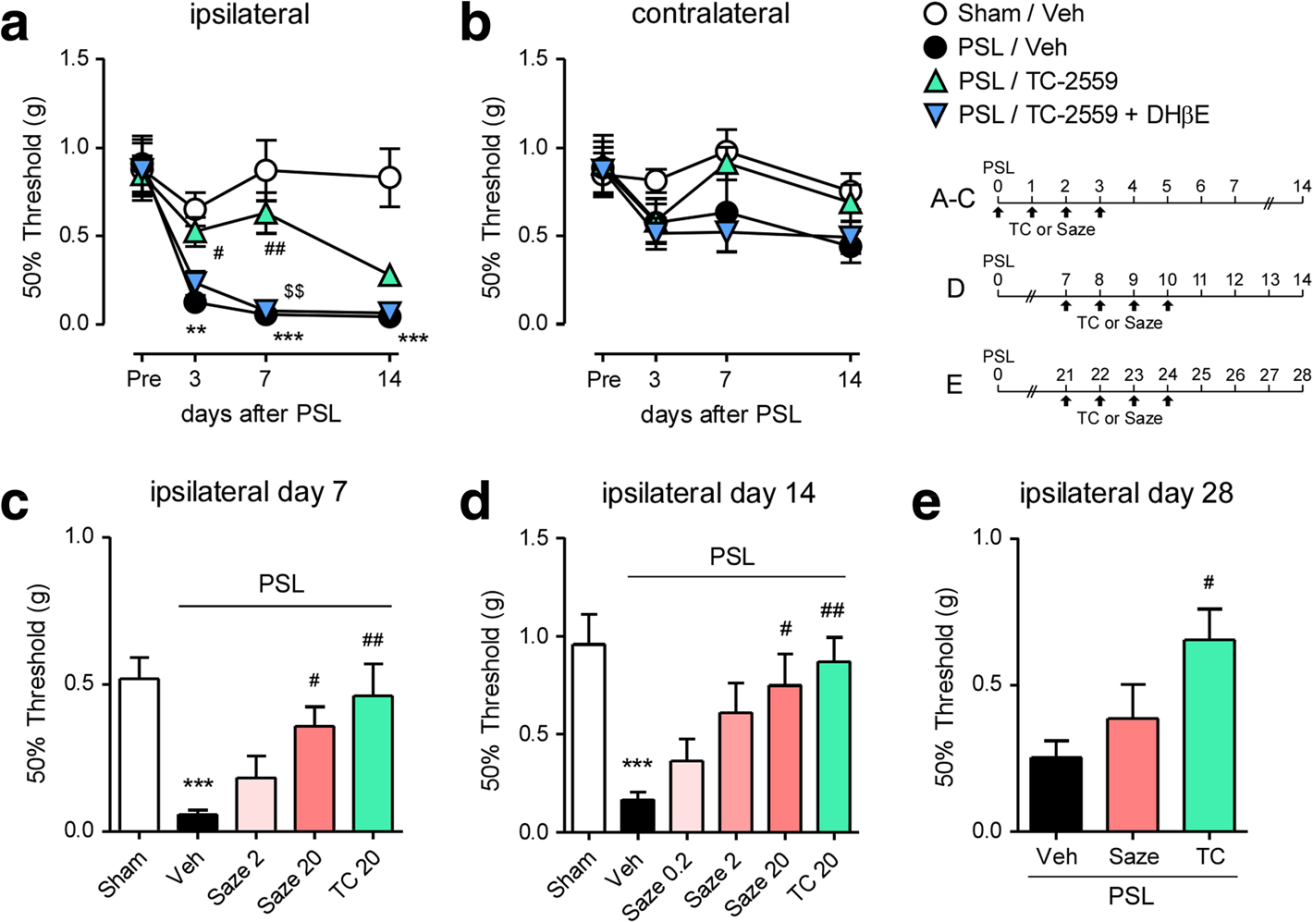
Improvement of mechanical allodynia after PSL by peripheral nicotinic acetylcholine receptor (nAChR) agonists. Mice were subject to PSL or sham surgery, and TC-2559 (20 nmol), sazetidine A (0.2–20 nmol), and DHβE (40 nmol) were p.n. administered four times according to the indicated schedules. The 50% paw withdrawal mechanical threshold was assessed by the up–down method using the von Frey test. **a** Attenuation of PSL-induced mechanical allodynia in the ipsilateral side by TC-2559 (days 0, 1, 2, and 3) are shown. Effects of TC-2559 were antagonized by the co-administration of DHβE. **b** There was no significant difference in the contralateral side in all groups. **c** Suppressive effects of TC-2559 and sazetidine A (days 0, 1, 2, and 3) on PSL-induced mechanical allodynia on day 7 in the ipsilateral side. **d** Improvement of PSL-induced mechanical allodynia on day 14 in the ipsilateral side by TC-2559 and sazetidine A (days 7, 8, 9, and 10) are shown. **e** Relief of PSL-induced mechanical allodynia on day 28 in the ipsilateral side by TC-2559 (days 21, 22, 23, and 24) are shown. Data are presented as the mean ± SEM; *n* = 5–11. ***P<0.001, **P<0.01 versus sham. ^##^*P* < 0.01, **P<0.01^#^*P* < 0.05 versus PSL/Veh, ^$$^*P*<0.01 versus PSL/TC-2559

Next, we examined whether peripheral administration of TC-2559 and another nAChR ligand, sazetidine A, affect neuropathic pain following PSL. On the ipsilateral side, mechanical allodynia defined by the reduction of 50% withdrawal threshold using the von Frey test was observed after PSL compared with sham surgery. PSL-induced mechanical allodynia was significantly prevented by p.n. administration of TC-2559 (20 nmol, days 0–3), and this effect was antagonized by co-administration of DHβE, an α4β2 nAChR antagonist (40 nmol, days 0–3; Fig. 4a). There was no significant difference of the 50% withdrawal threshold on the contralateral side (Fig. 4b). Then, we confirmed that preventive effect of p.n. administration of TC-2559 on PSL-induced mechanical allodynia is associated with the inhibition of inflammatory macrophages in the injured SCN. The mRNA levels of IRF5, a key transcription factor for inflammatory macrophages, were upregulated in the injured SCN for more than 2 weeks after PSL (Additional file 1: Figure S1A). The protein expression of IRF5 was also increased in the injured SCN on day 7 after PSL, and it was mainly colocalized with F4/80^+^ macrophages. Upregulation of IRF5 in the injured SCN after PSL was suppressed by p.n. administration of TC-2559 (20 nmol, days 0–3), and the effect was antagonized by co-administration of DHβE (40 nmol, days 0–3; Additional file 1: Figure S1B). Similar to TC-2559, p.n. administration of sazetidine A (2, 20 nmol, days 0–3) attenuated PSL-induced mechanical allodynia on day 7 (early phase; Fig. 4c). Furthermore, p.n. administration of TC-2559 (20 nmol, days 7–10) and sazetidine A (0.2–20 nmol, days 7–10) also relieved mechanical allodynia on day 14 after PSL when given during the middle/late phase (Fig. 4d). Notably, TC-2559 (20 nmol) also effectively attenuated mechanical allodynia on day 28 by p.n. administration during the much later phase of PSL (days 21–24; Fig. 4e). In addition, we examined whether the preventive effects of α4β2 nAChR agonists were mimicked by reducing activity of primary afferents using local anesthetic. The p.n. administration of bupivacaine (0.5% *w*/*v*; days 0–5) significantly prevented PSL-induced mechanical allodynia on day 7 (Additional file 2: Figure S2).

### Suppression of microglial activation in the SDH after PSL by peripheral TC-2559

**Fig. 5:**
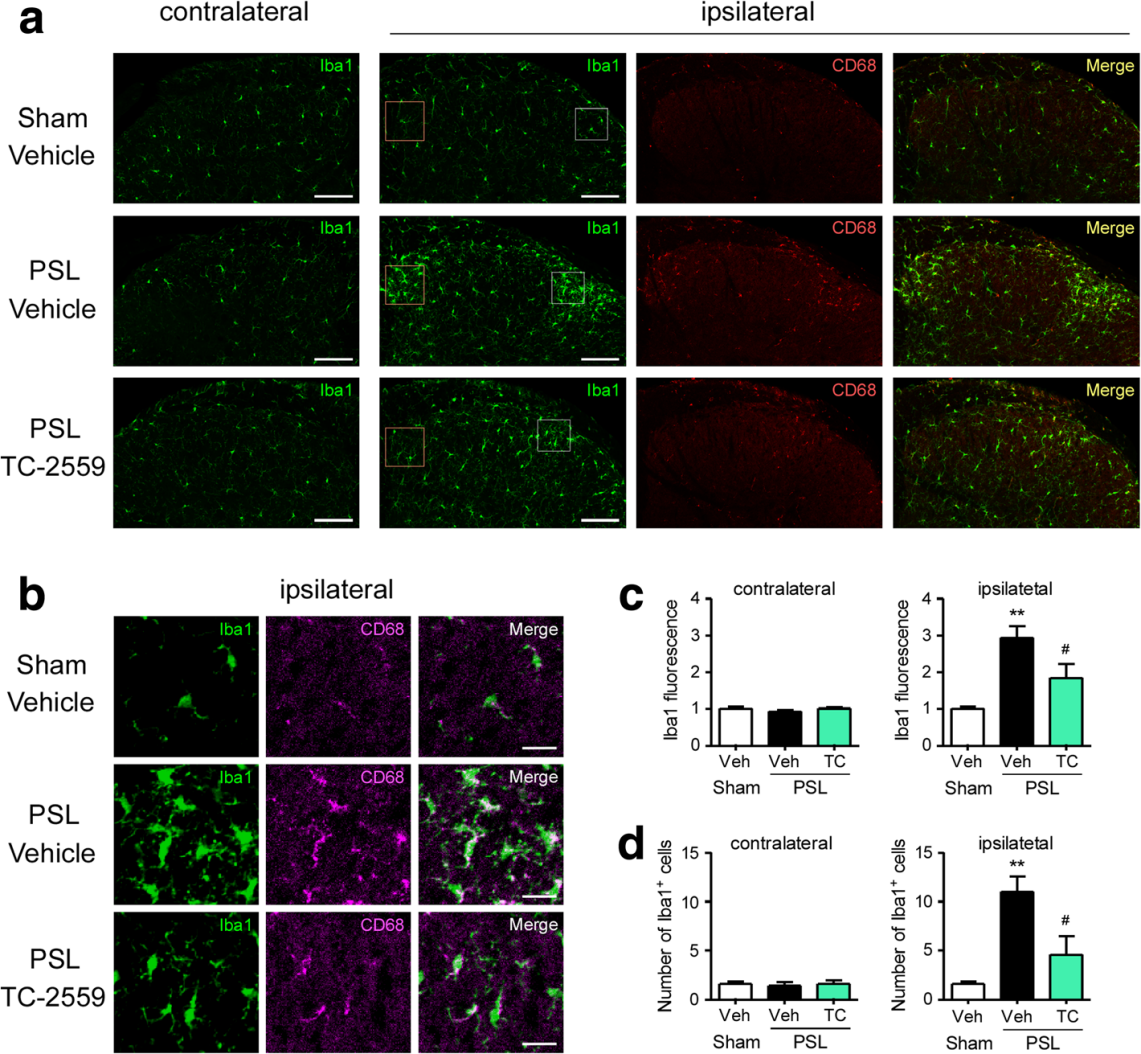
Suppression of microglial activation in the SDH after PSL by peripheral TC-2559. Mice were subject to PSL or sham surgery. TC-2559 was administered (p.n., 20 nmol) on days 0, 1, 2, and 3, and the lumbar spinal cord (L4–5) was collected. Expression of Iba1 and CD68 in the spinal dorsal horn (SDH) on day 7 after PSL was analyzed by immunohistochemistry. **a** Representative micrographs of Iba1 in the contralateral side and Iba1, CD68 and merged images in the ipsilateral sides of the SDH are presented as lower magnification. Scale bars = 100 μm. **b** Representative micrographs of Iba1, CD68 and merged images are presented as higher magnification from the square in the ipsilateral SDH (a; lateral area). Scale bars = 20 μm. Quantitative analyses of Iba1 fluorescence intensity (**c**) and the number of Iba1^+^ microglia (**d**) within the square of 100 × 100 μm^2^ (**a**; medial area) in the contralateral and ipsilateral SDH on day 7 after PSL are shown. Data are presented as the mean ± SEM; *n* = 5. **P < 0.01 versus sham. ^#^*P* < 0.05 versus PSL/Veh

To determine the relationship between peripheral macrophages and spinal microglia, we evaluated the morphological activation of microglia in the lumbar SDH (L4–5) after PSL with or without p.n. administration of TC-2559. Immunohistochemistry analysis revealed Iba1 immunoreactivity was markedly increased in the SDH of the ipsilateral side, but not the contralateral side, on day 7 after PSL in comparison with sham surgery (Fig. 5a). Inflammatory microglia-dominant molecule CD68 was also increased in the ipsilateral SDH after PSL, and it was colocalized with Iba1 (Fig. 5b). Upregulation of both Iba1 and CD68 in the ipsilateral SDH after PSL were significantly suppressed by p.n. administration of TC-2559 (days 0–3; Fig. 5a, b). Quantitative analysis revealed that the intensity of Iba1 fluorescence and the number of Iba1^+^ microglia were increased in the ipsilateral SDH on day 7 after PSL, and both were significantly attenuated by p.n. administration of TC-2559 (Fig. 5c, d).

### Effects of peripheral nAChR agonists on upregulation of microglial molecules in the SDH after PSL

**Fig. 6:**
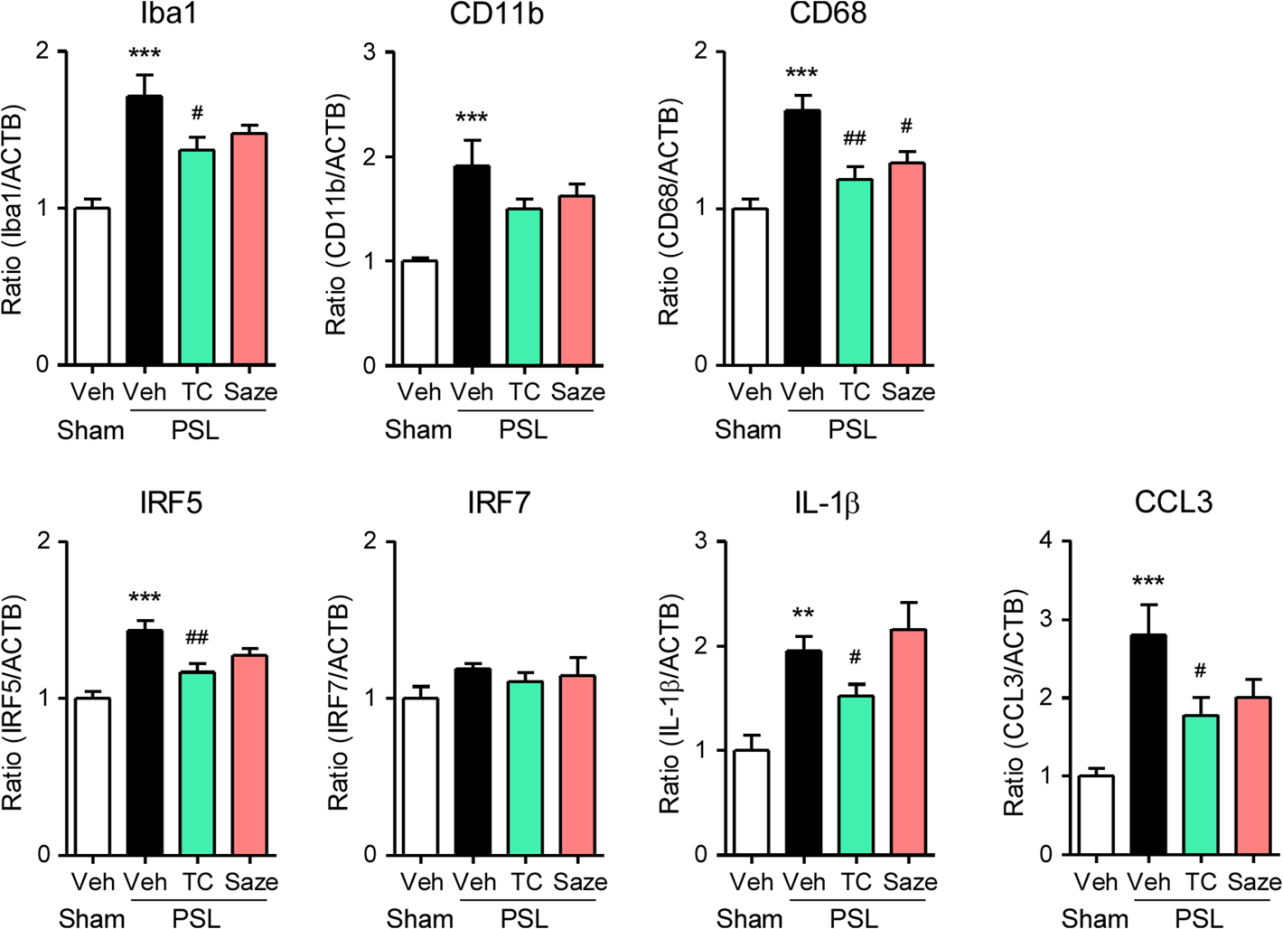
Suppressive effects of peripheral nAChR agonists on upregulation of microglial molecules in the SDH after PSL. Mice were subject to PSL or sham surgery. TC-2559 or sazetidine A was administered (p.n., 20 nmol) on days 0, 1, 2, and 3, and the lumbar SDH (L4–5) was collected. Expression levels of Iba1, CD11b, CD68, IRF5, IRF7, IL-1β, and CCL3 mRNA on day 7 after PSL were analyzed by RT-qPCR. Data are presented as the mean ± SEM; *n* = 6–7. ***P < 0.001, **P < 0.01 versus sham. ^##^*P* < 0.01, ^#^*P* < 0.05 versus PSL/Veh

**Fig. 7:**
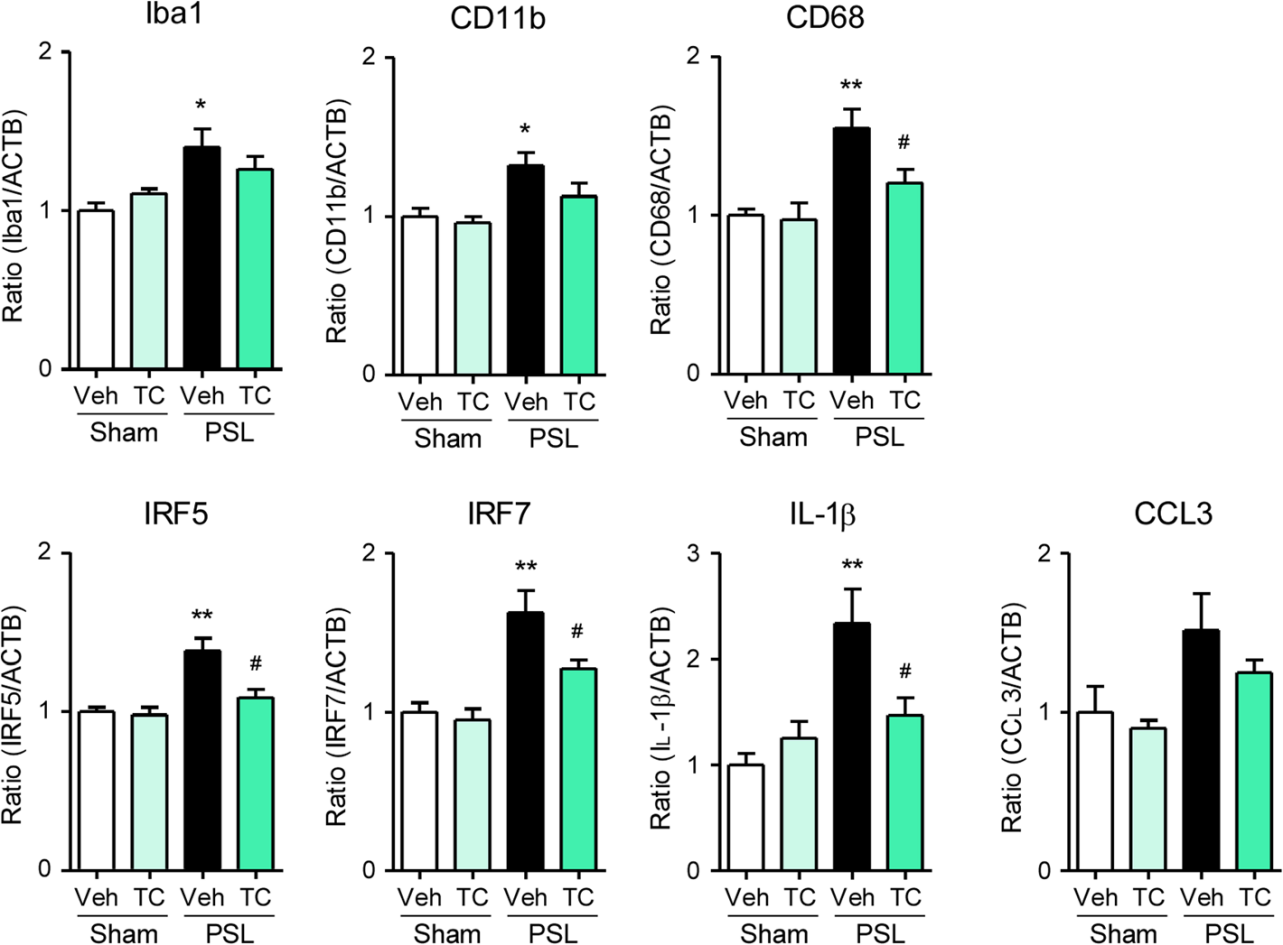
Relieving effects of peripheral TC-2559 on upregulation of microglial molecules in the SDH after PSL. Mice were subject to PSL or sham surgery. TC-2559 was administered (p.n., 20 nmol) on days 7, 8, 9, and 10, and the lumbar SDH (L4–5) was collected. Expression levels of Iba1, CD11b, CD68, IRF5, IRF7, IL-1β, and CCL3 mRNA on day 14 after PSL were analyzed by RT-qPCR. Data are presented as the mean ± SEM; *n* = 4–8. **P < 0.01, *P < 0.05 versus sham. ^#^*P* < 0.05 versus PSL/Veh

To confirm that peripheral nAChR agonists suppressed microglial activation, we evaluated mRNA expression levels of inflammatory microglia-dominant molecules by RT-qPCR. On day 7 after PSL, microglial markers (Iba1 and CD11b), inflammatory microglial factors (CD68 and IRF5), and soluble inflammatory factors (IL-1β and CCL3) were upregulated in the ipsilateral lumbar SDH (L4–5). There was no difference in the degree of these upregulation between L3–4 and L4–5 segments (Additional file 3: Figure S3). The p.n. administration of TC-2559 (20 nmol, days 0–3) significantly ameliorated the upregulation of these markers, whereas sazetidine A (20 nmol, days 0–3) partially attenuated the upregulation, which was consistent with behavioral experiments (Fig. 6). The protein expression of IRF5 was increased in the ipsilateral SDH on day 7 after PSL, and it was suppressed by the p.n. administration of TC-2559 (20 nmol, days 0–3; Additional file 4: Figure S4). On the other hand, the s.c. administration of TC-2559 (22.8 μmol/kg, days 0–3) also attenuated the mRNA expression level of Iba1 in the ipsilateral SDH on day 7 after PSL (Additional file 5: Figure S5). Furthermore, on day 14 after PSL, Iba1, CD11b, CD68, IRF5, IRF7, and IL-1β were upregulated in the ipsilateral SDH. The p.n. administration of TC-2559 (20 nmol, days 7–10) during the middle/late phase suppressed the upregulation of CD68, IRF5, IRF7, and IL-1β after PSL. TC-2559 had no effect on the mRNA levels of these molecules in the ipsilateral SDH on day 14 after sham surgery (Fig. 7).

## Discussion

This study provides three novel findings indicating that pharmacological inhibition of inflammatory macrophages by α4β2 nAChR agonists improves neuropathic pain elicited by peripheral nerve injury. First, systemic (s.c.) administration of TC-2559 during either the early or middle/late phase of PSL improved mechanical allodynia. Second, like TC-2559, local (p.n.) administration of sazetidine A during either the early phase or middle/late phase of PSL suppressed mechanical allodynia. Third and most importantly, p.n. administration of TC-2559 suppressed not only peripheral macrophages but also microglial activation in the SDH induced by peripheral nerve injury.

Profiles of infiltrating immune cells in the injured SCN after PSL have been mainly characterized by using histological procedures [11, 14, 15]. Notably, neutrophils, macrophages, and lymphocytes, which are well-characterized as regulators of neuropathic pain, increase in the injured SCN in distinct time courses after PSL. Neutrophils are most abundant in the early phase and decrease in the middle/late phase of PSL, whereas lymphocytes are hardly observed in the early phase and gradually increase from the middle phase [5, 21, 37]. In contrast, macrophages are clearly observed from the early phase to the middle/late phase of PSL, implying that macrophages orchestrate long-lasting neuroinflammation in the injured SCN, which underlies peripheral sensitization in neuropathic pain [7, 8, 19]. Here, we first demonstrated the accumulation of CD11b^+^ cells (peripheral leukocytes) in the injured SCN after PSL, analyzed by flow cytometry. Our results showed that the proportion of both CD11b^+^ cells and F4/80^+^ cells were greatest in the injured SCN on day 1, and that significant increases persisted for more than 2 weeks. The percentage of F4/80^+^ macrophages was approximately 30% of CD11b^+^ cells on day 1 and increased to more than 50% of CD11b^+^ cells during days 3 to 14 of PSL. Because CD11b is expressed on a variety of leukocytes [39–41], we hypothesized that neutrophils may account for the majority of infiltrating CD11b^+^ cells, consistent with a previous report [37]. After day 1, macrophages clearly accounted for the majority of accumulating immune cells, supporting the important role of macrophages in peripheral sensitization and neuropathic pain [22].

Infiltrating macrophages in the injured nerves induce an inflammatory phenotype, which expresses pain-enhancing molecules such as IL-1β, TNFα and CCL3 [16, 17, 20, 42]. Regarding the anti-inflammatory property of nAChR, α7 nAChR is most well-characterized, and administration of nicotine or selective α7 nAChR agonists attenuates various rodent models of inflammatory diseases through the downstream pathway of the α7 nAChR [26, 43, 44]. It has also been demonstrated that α4β2 nAChR exerts anti-inflammatory effects [45–47]. Moreover, we have previously reported that p.n. administration of nicotine acting on macrophages improves neuroinflammation and neuropathic pain through α4β2, but not α7, nAChR in mice, and p.n. administration of TC-2559 exerts significant suppressive effects on neuropathic pain after PSL [28, 29]. We have further clarified that inhibitory mechanisms of TC-2559 in inflammatory macrophages occur through the inhibition of signal transducer and activator of transcription 3 (STAT3), which is a key transcription factor for upregulation of inflammatory molecules (i.e., IL-1β and CCL3) in macrophages [48, 49]. Here, we determined that upregulation of IL-1β in the injured SCN, which reflects accumulation of inflammatory macrophages, was significantly attenuated by either systemic or local administration of TC-2559, and that LPS-induced upregulation of IL-1β in cultured peritoneal macrophages was also decreased by TC-2559. Moreover, upregulation of IRF5 localized on infiltrating macrophages was also suppressed by p.n. administration of TC-2559. These results indicate that α4β2 nAChR agonists, including TC-2559, can suppress inflammatory macrophages in vivo and in vitro. Therefore, we used α4β2 nAChR agonists as an inhibitor of inflammatory macrophages.

This is the first report showing that systemic administration of TC-2559 attenuates PSL-induced neuropathic pain. Consistent with our previous study using p.n. administration [29], s.c.-administered TC-2559 exerted a suppressive effect on PSL-induced mechanical allodynia in both the early and middle/late phases of PSL. Given that systemic administration of TC-2559 also suppressed upregulation of IL-1β expression in the injured SCN, systemic administration can be also used for pharmacological inhibition targeting inflammatory peripheral macrophages. There are several reports demonstrating that systemic administration of α4β2 nAChR agonists, including nicotine, epibatidine, and ABT-594, attenuates experimental inflammatory pain and neuropathic pain in rodents [50]. Unlike our study, these reports focused on the antinociceptive mechanisms via spinal or supraspinal actions. The α4β2 nAChR are expressed in the nucleus raphe magnus and locus coeruleus, which play a central role in descending inhibitory monoaminergic pathways, and other regions modulating endogenous pain-inhibitory pathways [51, 52]. Thus, systemically administered α4β2 nAChR agonists have been considered to attenuate abnormal pain transmission by supraspinal mechanisms. In addition, the α4β2 nAChR agonist activates spinal GABAergic inhibitory interneurons in the SDH, and intrathecal administration of α4β2 nAChR agonists improves neuropathic pain via enhancement of inhibitory mechanisms of the pain processing within the spinal cord [53]. Recently, Cheng et al. have reported that intraperitoneal administration of TC-2559 (3–10 mg/kg) attenuates inflammatory pain by formalin injection and chronic constriction injury-induced neuropathic pain [54]. These effects of TC-2559 were explained by the activation of inhibitory synaptic transmission in the SDH [54]. Nonetheless, given that the effective dose of TC-2559 was similar to our results, it may be possible that there are common mechanisms, at least in part, for suppressive effects of TC-2559 in different pain models.

In addition to nicotine and TC-2559, as we have previously demonstrated [28, 29], we found that p.n. administration of sazetidine A during either the early or middle/late phase of PSL attenuated mechanical allodynia. However, the effectiveness of sazetidine A was partial and weaker than that of TC-2559. In particular, sazetidine A did not have an effect on mechanical allodynia in the much later phase (days 21–24) of PSL, whereas TC-2559 administration during this phase was able to relieve mechanical allodynia. The functional gap between TC-2559 and sazetidine A might be explained by the efficacies of these two compounds for α4β2 nAChR. In comparison with TC-2559 [55, 56], sazetidine A is characterized as a partial agonist, which has limited efficacy [57–59], and it may only partially activate intracellular signaling underlying anti-inflammatory properties of α4β2 nAChR on macrophages. As nicotine and TC-2559 are full agonists, it is appropriate that the effects of sazetidine A are weaker and limited compared with these agents. Nonetheless, these lines of evidence provide important insight; although α4β2 nAChR are an attractive pharmacological target of neuropathic pain driven by inflammatory macrophages, a compound with full agonist property (or sufficient efficacy) is required to obtain ideal relieving effects for neuropathic pain.

It is pivotal that microglia are activated after peripheral nerve injury and largely contribute to pathogenesis of neuropathic pain. Activation of microglia is regulated by a variety of neurotransmitters, neuropeptides, cytokines, and chemokines [5, 33–35]. Despite numerous reports demonstrating the functional significance of microglia in spinal regulation of neuropathic pain, the controlling mechanisms and therapeutic potential for targeting microglia are still controversial. Clinical evidence has shown that peripheral neuropathic pain is maintained by prolonged activity of primary afferents [32]. Indeed, such notion is consistent with our result that reduction of ectopic activity of primary afferent by the p.n. administration of bupivacaine prevented PSL-induced neuropathic pain. Given that inflammatory cytokines and chemokines derived from macrophages directly enhance ectopic activity of primary afferents causing peripheral sensitization [8, 13, 60], it is worth understanding whether macrophage-driven peripheral sensitization correlates with microglial activation in the SDH following nerve injury. We found that inhibition of inflammatory macrophages by p.n.-administered TC-2559 suppressed microglial activation in the SDH evaluated by Iba1 expression, suggesting a novel relationship between inflammatory peripheral macrophages and spinal microglia. Moreover, TC-2559 attenuated the upregulation of general microglial markers (Iba1 and CD11b) and inflammatory microglia-dominant molecules (CD68, IRF5, IRF7, IL-1β, and CCL3) [61–64], which are characterized as pain-regulatory factors under the spinal sensitization. Compared with TC-2559, the attenuating effects of sazetidine A on the upregulation of microglial molecules in the SDH were weaker and partial, which was consistent with the behavioral outcomes as a result of its partial agonist property. Most importantly, we found that peripheral administration of TC-2559 on days 7 to 10 significantly decreased the PSL-induced upregulation of microglial factors in the SDH on day 14. These findings reveal the novel regulatory mechanisms of spinal microglia by macrophage-driven peripheral neuroinflammation and emphasize the therapeutic potential for targeting macrophages. Future studies are needed to determine the key components maintaining microglial activation derived from primary afferents with abnormal activity.

## Conclusions

Collectively, this is the first study demonstrating that pharmacological inhibition of inflammatory macrophages by using α4β2 nAChR agonists can improve neuropathic pain by either systemic or local administration in mice. Notably, TC-2559, a full agonist of α4β2 nAChR, exhibited a wide therapeutic window on neuropathic pain after nerve injury, and its effectiveness might be explained by dual inhibition of peripheral macrophages and spinal microglia, which are key components of neuropathic pain. Given that the pathogeneses of diverse types of neuropathic pain are at least in part associated with inflammatory macrophages [19], pharmacological inhibition of inflammatory macrophages could be nominated as a novel approach to resolve the limitations of current therapies. Future mechanistic and pharmacological studies focusing on inflammatory macrophage-driven neuroinflammation may generate a novel pharmacotherapy to relieve intractable pain.

## Additional files

Additional file 1: **Figure S1.** Inhibition of interferon regulatory factor 5 (IRF5) upregulation in macrophages by TC-2559. Mice were subject to PSL or sham surgery. TC-2559 (20 nmol) and DHβE (40 nmol) were perineurally (p.n.) administered on days 0, 1, 2, and 3, and the SCN was collected. (A) Time course of IL-1β mRNA expression in the SCN on days 1 to 14 after sham or PSL was analyzed by RT-qPCR. Data are presented as the mean ± SEM; *n* = 6–7. ***P < 0.001 versus Sham. (B) Representative micrographs of F4/80, IRF5, and merged images in the injured SCN on day 7 after PSL analyzed by immunohistochemistry are presented. Scale bars = 20 μm. (TIFF 7197 kb)

Additional file 2: **Figure S2.** Improvement of mechanical allodynia after PSL by peripheral bupivacaine. Mice were subject to PSL or sham surgery, and bupivacaine (Bupi; 0.5% *w*/*v*) was p.n. administered once a day for 6 days (days 0–5). The 50% paw withdrawal mechanical threshold was assessed by the up-down method using the von Frey test. Improving effects of bupivacaine on PSL-induced mechanical allodynia on day 7 in the ipsilateral side are shown. Data are presented as the mean ± SEM; *n* = 6–7. ***P < 0.001 versus sham. ^#^*P* < 0.05 versus PSL/Veh. (TIFF 1190 kb)

Additional file 3: **Figure S3.** Upregulation of microglial molecules in the SDH after PSL. Mice were subject to PSL or sham surgery, and the L3–4 and L4–5 segments of lumbar SDH were separately collected. Expression levels of Iba1 and CD11b mRNA in each segment on day 7 after PSL were analyzed by RT-qPCR. Data are presented as the mean ± SEM; *n* = 5–6. **P < 0.01, *P < 0.05 versus sham. (TIFF 2362 kb)

Additional file 4: **Figure S4.** Suppressive effects of peripheral TC-2559 on upregulation of IRF5 in the SDH after PSL. Mice were subject to PSL or sham surgery. TC-2559 was administered (p.n., 20 nmol) on days 0, 1, 2, and 3, and the lumbar SDH (L4–5) was collected. Expression level of IRF5 protein on day 7 after PSL was analyzed by western blotting. Data are presented as the mean ± SEM; *n* = 5–6. **P < 0.01 versus sham. ^#^*P* < 0.05 versus PSL/Veh. (TIFF 898 kb)

Additional file 5: **Figure S5.** Suppressive effects of systemic TC-2559 on upregulation of Iba1 in the SDH after PSL. Mice were subject to PSL. TC-2559 was administered (s.c., 22.8 μmol/kg) on days 0, 1, 2, and 3, and the lumbar SDH (L4–5) was collected. Expression levels of Iba1 and CD11b mRNA on day 7 after PSL were analyzed by RT-qPCR. Data are presented as the mean ± SEM; *n* = 5–7. ^#^*P* < 0.05 versus PSL/Veh. (TIFF 1232 kb)

## Abbreviations

ACTB: β-Actin
CCL: CC-chemokine ligand
DHβE: Dihydro-β-erythroidine hydrobromide ((2*S*,13b*S*)-2-Methoxy-2,3,5,6,8,9,10,13-octahydro-1*H*,12*H*-benzo[*i*]pyrano[3,4-*g*]indolizin-12-one hydrobromide)
DMEM: Dulbecco’s modified Eagle’s medium
FBS: Fetal bovine serum
GAPDH: Glyceraldehyde-3-phosphate dehydrogenase
IL-1β: Interleukin-1β
IRF: Interferon regulatory factor
LPS: Lipopolysaccharide
nAChR: Nicotinic acetylcholine receptor
p.n.: Perineural
P/S: Penicillin–streptomycin
PBS: Phosphate-buffered saline
PSL: Partial sciatic nerve ligation
s.c.: Subcutaneous
sazetidine A: 6-[5-[(*2S*)-2-Azetidinylmethoxy]-3-pyridinyl]-5-hexyn-1-ol dihydrochloride
SCN: Sciatic nerve
SDH: Spinal dorsal horn
TBS: Tris-buffered saline
TC-2559: 4-(5-ethoxy-3-pyridinyl)-*N*-methyl-(*3E*)-3-buten-1-amine difumarate
TNFα: Tumor necrosis factor-α
