# Supplementary figures and images for: Inhibition of peripheral macrophages by nicotinic acetylcholine receptor agonists suppresses spinal microglial activation and neuropathic pain in mice with peripheral nerve injury
Source: J Neuroinflammation. 2018 Mar 27;15:96. doi: 10.1186/s12974-018-1133-5 (PMC5872578; doi:10.1186/s12974-018-1133-5)

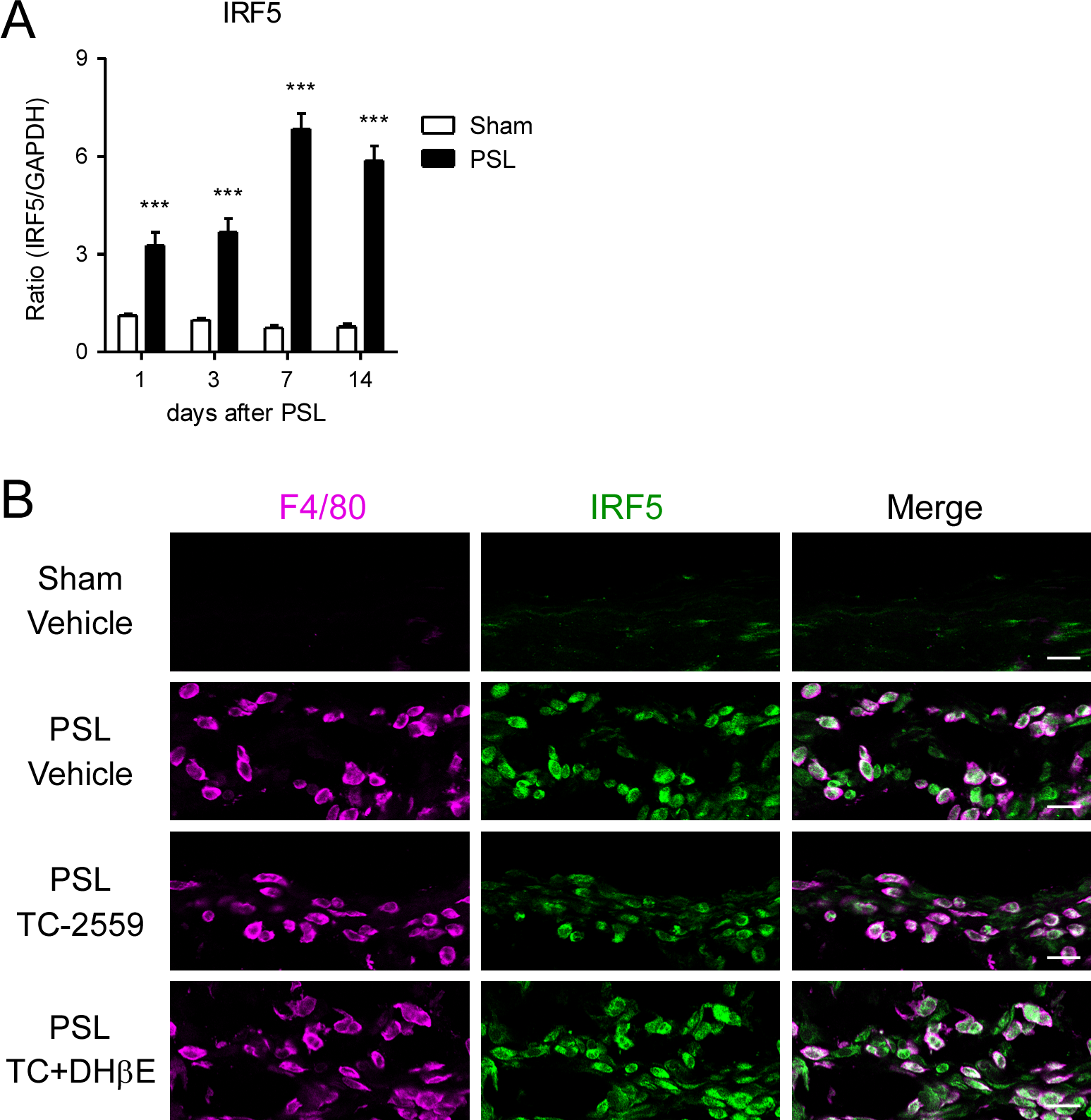

Supplement: Supplementary file 1 — Figure S1. Inhibition of interferon regulatory factor 5 (IRF5) upregulation in macrophages by TC-2559. Mice were subject to PSL or sham surgery. TC-2559 (20 nmol) and DHβE (40 nmol) were perineurally (p.n.) administered on days 0, 1, 2, and 3, and the SCN was collected. (A) Time course of IL-1β mRNA expression in the SCN on days 1 to 14 after sham or PSL was analyzed by RT-qPCR. Data are presented as the mean ± SEM; n = 6–7. ***P < 0.001 versus Sham. (B) Representative micrographs of F4/80, IRF5, and merged images in the injured SCN on day 7 after PSL analyzed by immunohistochemistry are presented. Scale bars = 20 μm. (TIFF 7197 kb) [file 12974_2018_1133_MOESM1_ESM.tif]

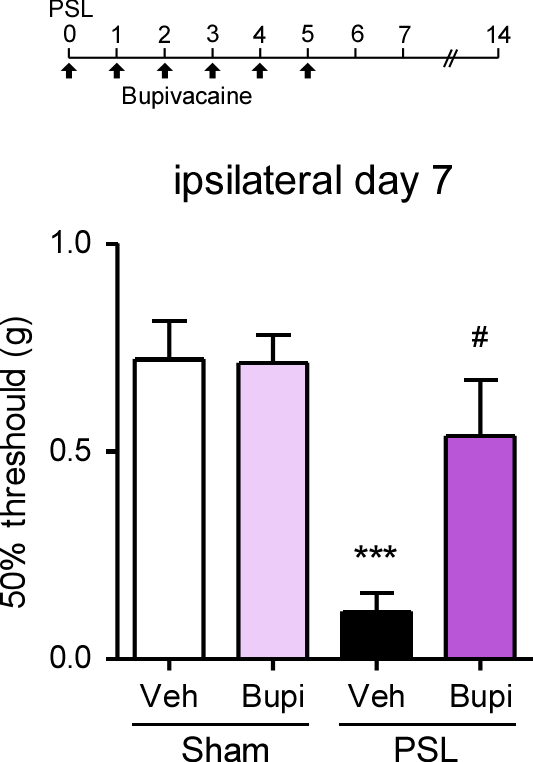

Supplement: Supplementary file 2 — Figure S2. Improvement of mechanical allodynia after PSL by peripheral bupivacaine. Mice were subject to PSL or sham surgery, and bupivacaine (Bupi; 0.5% w/v) was p.n. administered once a day for 6 days (days 0–5). The 50% paw withdrawal mechanical threshold was assessed by the up-down method using the von Frey test. Improving effects of bupivacaine on PSL-induced mechanical allodynia on day 7 in the ipsilateral side are shown. Data are presented as the mean ± SEM; n = 6–7. ***P < 0.001 versus sham. #P < 0.05 versus PSL/Veh. (TIFF 1190 kb) [file 12974_2018_1133_MOESM2_ESM.tif]

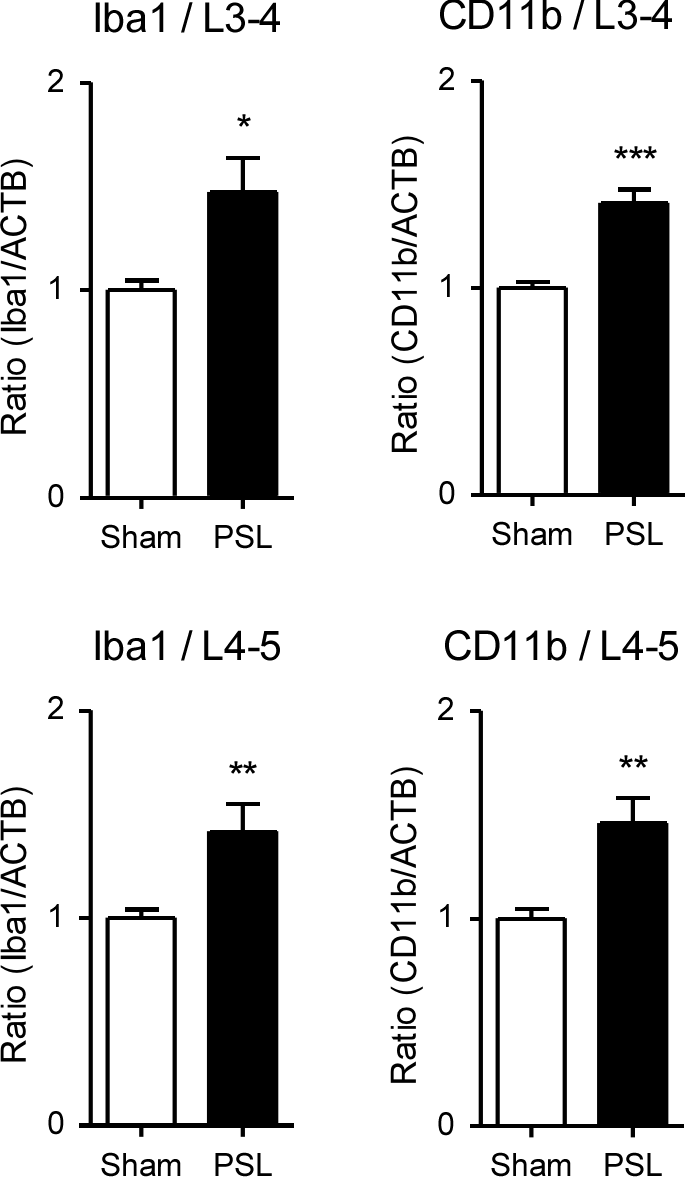

Supplement: Supplementary file 3 — Figure S3. Upregulation of microglial molecules in the SDH after PSL. Mice were subject to PSL or sham surgery, and the L3–4 and L4–5 segments of lumbar SDH were separately collected. Expression levels of Iba1 and CD11b mRNA in each segment on day 7 after PSL were analyzed by RT-qPCR. Data are presented as the mean ± SEM; n = 5–6. **P < 0.01, *P < 0.05 versus sham. (TIFF 2362 kb) [file 12974_2018_1133_MOESM3_ESM.tif]

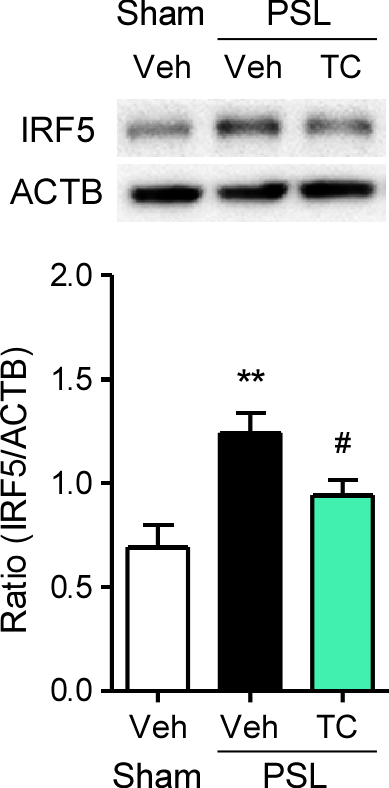

Supplement: Supplementary file 4 — Figure S4. Suppressive effects of peripheral TC-2559 on upregulation of IRF5 in the SDH after PSL. Mice were subject to PSL or sham surgery. TC-2559 was administered (p.n., 20 nmol) on days 0, 1, 2, and 3, and the lumbar SDH (L4–5) was collected. Expression level of IRF5 protein on day 7 after PSL was analyzed by western blotting. Data are presented as the mean ± SEM; n = 5–6. **P < 0.01 versus sham. #P < 0.05 versus PSL/Veh. (TIFF 898 kb) [file 12974_2018_1133_MOESM4_ESM.tif]

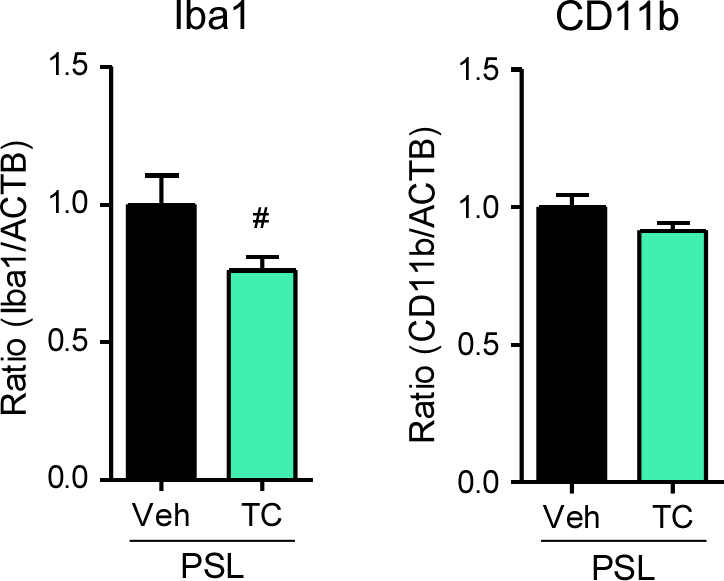

Supplement: Supplementary file 5 — Figure S5. Suppressive effects of systemic TC-2559 on upregulation of Iba1 in the SDH after PSL. Mice were subject to PSL. TC-2559 was administered (s.c., 22.8 μmol/kg) on days 0, 1, 2, and 3, and the lumbar SDH (L4–5) was collected. Expression levels of Iba1 and CD11b mRNA on day 7 after PSL were analyzed by RT-qPCR. Data are presented as the mean ± SEM; n = 5–7. #P < 0.05 versus PSL/Veh. (TIFF 1232 kb) [file 12974_2018_1133_MOESM5_ESM.tif]
